# Nitrification mainly driven by ammonia-oxidizing bacteria and nitrite-oxidizing bacteria in an anammox-inoculated wastewater treatment system

**DOI:** 10.1186/s13568-021-01321-6

**Published:** 2021-11-27

**Authors:** Jing Lu, Yiguo Hong, Ying Wei, Ji-Dong Gu, Jiapeng Wu, Yu Wang, Fei Ye, Jih-Gaw Lin

**Affiliations:** 1 Key Laboratory for Water Quality and Conservation of the Pearl River Delta, Ministry of Education, Institute of Environmental Research at Greater Bay, Guangzhou University, Guangzhou, 510006 People’s Republic of China; 2 School of Environmental Science and Engineering, Guangzhou University, Guangzhou, China; 3 Environmental Engineering, Guangdong Technion Israel Institute of Technology, 241 Daxue Road, Shantou, 515063 Guangdong China; 4 Institute of Environmental Engineering, National Chiao Tung University, 1001 University Road, Hsinchu City, 30010 Taiwan

**Keywords:** Nitrification, Anammox-inoculated WWTPs, Ammonia-oxidizing bacteria (AOB), Ammonia-oxidizing archaea (AOA), *Nitrospira*

## Abstract

Anaerobic ammonium oxidation (anammox) process has been acknowledged as an environmentally friendly and time-saving technique capable of achieving efficient nitrogen removal. However, the community of nitrification process in anammox-inoculated wastewater treatment plants (WWTPs) has not been elucidated. In this study, ammonia oxidation (AO) and nitrite oxidation (NO) rates were analyzed with the incubation of activated sludge from Xinfeng WWTPs (Taiwan, China), and the community composition of nitrification communities were investigated by high-throughput sequencing. Results showed that both AO and NO had strong activity in the activated sludge. The average rates of AO and NO in sample A were 6.51 µmol L^−1^ h^−1^ and 6.52 µmol L^−1^ h^−1^, respectively, while the rates in sample B were 14.48 µmol L^−1^ h^−1^ and 14.59 µmol L^−1^ h^−1^, respectively. The abundance of the nitrite-oxidizing bacteria (NOB) *Nitrospira* was 0.89–4.95 × 10^11^ copies/g in both samples A and B, the abundance of ammonia-oxidizing bacteria (AOB) was 1.01–9.74 × 10^9^ copies/g. In contrast, the abundance of ammonia-oxidizing archaea (AOA) was much lower than AOB, only with 1.28–1.53 × 10^5^ copies/g in samples A and B. The AOA community was dominated by *Nitrosotenuis*, *Nitrosocosmicus,* and *Nitrososphaera,* while the AOB community mainly consisted of *Nitrosomonas* and *Nitrosococcus*. The dominant species of *Nitrospira* were *Candidatus* Nitrospira defluvii, *Candidatus* Nitrospira Ecomare2 and *Nitrospira inopinata*. In summary, the strong nitrification activity was mainly catalyzed by AOB and *Nitrospira*, maintaining high efficiency in nitrogen removal in the anammox-inoculated WWTPs by providing the substrates required for denitrification and anammox processes.

**Supplementary Information:**

The online version contains supplementary material available at 10.1186/s13568-021-01321-6.

## Introduction

The discharge of nitrogen containing wastewater into natural waters has become a global concern, resulting in problems such as acidification and eutrophication (Canfield et al. 2010; Kuypers et al. 2018; Bailes et al. 2020; Lan et al. 2011). Thus, optimizing the performance of nitrogen removal in WWTPs is essential in order to control water pollution caused by the release of anthropogenic waste. Biological nitrification and denitrification procedures have been widely applied to ammonium-rich WWTPs (Lan et al. 2011; Ali and Okabe 2015) in which ammonia is firstly transformed to nitrate via nitrification, with nitrate then converted to nitrogen gas by anoxic biotransformation using organic carbon as an electron source (Yang et al. 2020a, b). However, biological nitrification and denitrification is not the most effective process in nitrogen removal owing to the high oxygen demand of complete nitrification, the heterotrophic denitrification requires an external carbon source, accompanied by the production of sludge and nitrous oxide (N_2_O) (Shalini et al. 2012; Wang et al. 2018; Xin et al. 2020).

The increasing need to reduce the consumption of energy and resources by treatment systems has contributed to the development of several novel nitrogen removal techniques, such as a single reactor system for high ammonium removal over nitrite (SHARON) (Hellinga et al. 1998; Dongen et al. 2001), completely autotrophic nitrogen removal over nitrite (CANON) (Sliekers et al. 2002), oxygen-limited autotrophic nitrification–denitrification (OLAND) (Kuai et al. 1998), and anaerobic ammonium oxidation (anammox) (Jetten et al. 1998). In particular, the anammox procedure has been proven to be more efficient than conventional nitrification–denitrification process (Yang et al. 2020a, b; Ma et al. 2016; Wu et al. 2019; Bucci et al. 2020), achieving 60% reduction in oxygen demand (e.g., aeration), 90% reduction in sludge, 100% reduction in the demand for organic carbon sources and less or no N_2_O emission (Kartal et al. 2008; Kuenen 2008; Okabe et al. 2011; Van et al. 1995; Strous et al. 1999). The anammox reaction directly transforms ammonium and nitrite into nitrogen gas. The nitrite acts as an electron accepter in this process. (Kartal et al. 2013). However, due to the long doubling time and low typically biological yield of anammox bacteria in bioreactor systems, establishing anammox-inoculated WWTPs capable of effective inorganic nitrogen removal is time-consuming compared to conventional WWTP processes (Ali et al. 2015; Awata et al. 2013; Cho et al. 2011; Strous et al. 1998; van et al. 2007). In general, visible anammox bacterial granules are not observed until 6–9 months after the initial inoculation of WWTPs (Meng et al. 2017; Yang et al. 2018; Azari et al. 2017). The high-throughput sequencing technique was used to further revealing the composition of the main microorganism community in WWTPs (Yang et al. 2020a, b).

Previous researches on nitrogen removal in WWTPs focused on the denitrification and anammox processes, which transformed nitrate and nitrite to nitrogen gas (Ali and Okabe 2015; Yang et al. 2020a, b; Ali et al. 2015). In anammox-inoculated WWTPs, various types of microbial pathways play significant roles in the procedure of nitrogen transformation especially the anammox process occupy the main position. Denitrification is an important process for nitrogen removal while nitrification is an essential intermediate process transforming ammonia to nitrite or nitrate. However, despite nitrification being an essential and rate-limiting step in microbial nitrogen-cycling networks, the role of nitrification in anammox-inoculated WWTPs has not been comprehensively understood in current available researches (Cortés-Lorenzo et al. 2015; Wang et al. 2018; Xin et al. 2020; Egli et al. 2003; Zheng et al. 2017) and the community microbial activity of the major nitrifiers in anammox-driven WWTPs remain unknown. A more complete understanding of nitrification can increase the efficiency of nitrogen removal in anammox-inoculated WWTPs.

In this research, the microbial communities related to nitrification were studied in an anammox-inoculated WWTPs, including ammonia-oxidizing bacteria (AOB), ammonia-oxidizing archaea (AOA), and nitrite-oxidizing bacteria (NOB). The aims of this project were to (i) determine the potential nitrification rates (including rates of AO and NO); (ii) analyse the community composition of the key functional microbes contributing to nitrification in the wastewater treatment ecosystem; and (iii) understand the role of nitrification in the anammox-inoculated WWTPs.

## Materials and methods

### Description of the anammox-inoculated WWTPs and sample collection

The activated sludge samples used in this study were obtained from an anammox bacteria inoculated aeration tank in Xinfeng WWTPs in Taiwan, the northeast of China. The WWTPs were used for treating mixed wastewater including landfill leachate wastewater, car washing wastewater, and fertilizer wastewater (Yang et al. 2020a, b). Fresh activated sludge samples were collected from the aeration tank (sample A and sample B were collected on May 13th 2019 and October 12th 2019, respectively) using 500 mL polypropylene bottles, then stored with ice packs during the transportion to the laboratory. The collected samples were firstly divided into two parts. Then, one part of samples were stored in the refrigerator at 4 ℃ for rates analysis, the other part were stored in the refrigerator at − 20 ℃ for the analysis of community composition and abundance.

### Nitrification rate measurements

**Fig. 1:**
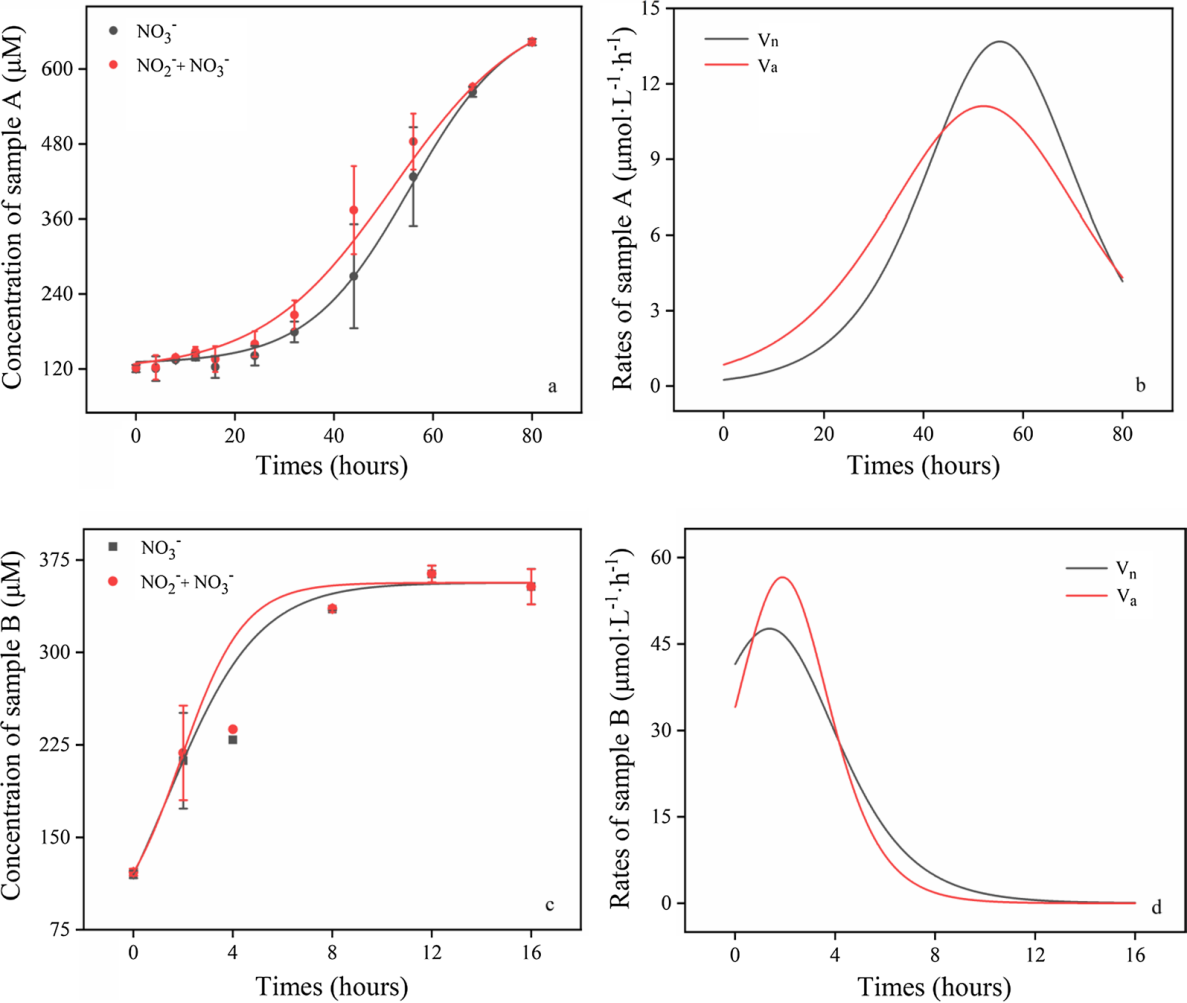
Boltzmann fitted curves (**a**, **c**) and differential curves (**b**, **d**) from Boltzmann fitting equations of the DIN time series for instantaneous rates of ammonia oxidation (Va) and nitrite oxidation (Vn) of the water samples

**Fig. 2:**
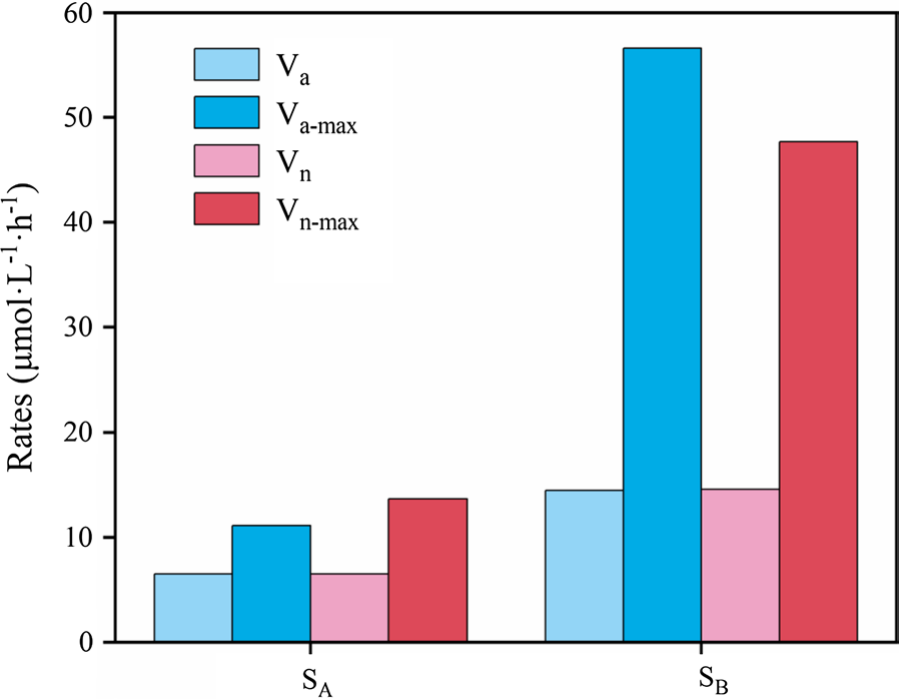
Average and maximum rates of ammonia oxidation and nitrite oxidation

The AO and NO rates were calculated using methods previously described by Hong (Hong et al. 2018). Briefly, the 250 mL sterile wide-mouth bottles were used to incubate samples (HDPE, Thermo Fisher Scientific, US) containing 1 g activated sludge slurry, which were then incubated at 28 ℃ with continual agitation at 75 rpm to maintain aerated conditions. To decrease dissolved inorganic nitrogen (DIN) assimilation by phytoplankton, samples were cultured in the dark. The incubation supernatant was sampled every 6 h for measurement of ammonia, nitrite, and nitrate concentrations. The previous research has suggested that mathematical modeling methods are more suitable for the analysis of nitrification rates because nitrification was a non-linear dynamic process (Hong et al. 2018). Large errors will introduce if nitrification rates are calculated via using only two or three data points to establish linear regression. Therefore, a mathematical modeling method was adopted in the present study (Fig. 1). Firstly, the corresponding mathematical equation was obtained by fitting a smoothed time series of DIN concentration using the Boltzmann distribution pattern. The fitted NO_x_^−^ curves were used to calculate the average rates of AO (V_a_, Eq. (1)) and NO (V_n,_ Eq. (2)). Then, instantaneous V_a_ and V_n_ equations and curves were calculated according to the first derivative of the fitting curves (Fig. 1). The average and instantaneous data parameters (including maximum instantaneous data points) were suggested in Fig. 2. The three indices (V_a_, V_n_ and their maximum value) described the average nitrification potential and maximum nitrification potential of a sample. The dynamic process of nitrification in samples was depicted in Fig. 1. The index of nitrogen balance (INB) was used to make an estimation with the total amount of variability of DIN in the incubation system (Eq. (3)).

1 $$\overline{{V_{a} }} = \frac{{\left[ {NO_{{_{3} }}^{ - } + NO_{2}^{ - } } \right]_{final} \_\left[ {NO_{3}^{ - } + NO_{2}^{ - } } \right]_{initial} }}{\Delta T}$$

2 $$\overline{{V_{n} }} = \frac{{\left[ {NO_{3}^{ - } } \right]_{final} - \left[ {NO_{3}^{ - } } \right]_{initial} }}{\Delta T}$$

3 $$INB = \frac{{DIN_{final} }}{{DIN_{initial} }} = \frac{{\left[ {NH_{4}^{ + } } \right]_{final} + \left[ {NO_{2}^{ - } } \right]_{final} + \left[ {NO_{3}^{ - } } \right]_{final} }}{{\left[ {NH_{4}^{ + } } \right]_{initial} + \left[ {NO_{2}^{ - } } \right]_{initial} + \left[ {NO_{3}^{ - } } \right]_{initial} }}$$

### DNA extraction and PCR amplification

Total DNA was extracted 0.3 g from each sample using a PowerSoil DNA isolation kit (Mo Bio, CA, US), then stored it in the refrigerator with temperature of − 80 °C for further analysis. The NanoDrop Lite spectrophotometer (Thermo Fisher Scientific, DE, US) was used to determine the concentration of extracted DNA. Using extracted DNA as a template, the respective primer pairs (Arch-amoAF/Arch-amoAR, amoA-1F/amoA-2R, Nxr-f27/Nxr-r617) were used to participate in the PCR amplification targeting archaeal *amoA*, bacterial *amoA*, and *nxrB genes* separately (Reddy et al. 2014; Rotthauwe et al. 1997; Hong et al. 2020). Optimized PCR was run using mixtures with a total amount of 25 μL, composed of 12.5 μL of GoTaq Green Master Mix (Promega, US), 1 μL of forward and reverse primers (15 μM), 1 μL of template DNA, and 9.5 μL of ddH_2_O. The thermocycler program for archaeal *amoA* and bacterial *amoA* was shown below: 95 °C for 3 min, followed by 35 cycles of 95 °C for 45 s, 53 °C for 45 s, and 72 °C for 45 s, finally followed by elongation at 72 °C for 10 min. The thermocycler program for *nxrB* was shown below: 95 °C for 2 min, followed by 32 cycles of 95 °C for 45 s, 62 °C for 45 s, and 72 °C for 45 s, finally followed by elongation at 72 °C for 5 min. The NanoDrop spectrophotometer (NanoDrop Technologies, DE, US) and MiniBEST agarose gel DNA extraction kit (TaKaRa, Beijing, China) were used to check the concentration of PCR products and purify the products respectively. Then, stored it in a single tube for high-throughput sequencing. Finally, the PCR products through purification were sequenced on the Illumina MiSeq platform.

### High-throughput sequencing analysis

High-throughput sequencing was performed according to the standard protocol described in previously studies using Mothur software v.1.40.5 (Schloss et al. 2009; Wu et al. 2018; He et al. 2016). Tags and primers were firstly removed from the obtained sequences. Then, the quality-trimmed sequences were aligned to the gene database including sequences for *amoA*-AOA (He 2018), *amoA-*AOB (The 16S rRNA gene sequences of the identified strains and uncultured sequences from environmental samples as seed sequences in database were obtained from NCBI and Fungene), and *nxrB* (Jiao et al. 2018). The *seqs*. command was applied to filter and remove badly aligned sequences, while the commands *pre.cluster, chimera.uchime* and *remove.seqs* were used to reduce errors and remove chimeric sequences. Operational taxonomic units (OTUs) were identified using 0.06, 0.13, and 0.06 cut off values for OTU clustering of AOA, AOB, and Nitrospira, respectively.

### Real-time quantitative PCR

The abundance of nitrifier prokaryotes was determined by real-time PCR (Q-PCR) using an Eco Real-Time PCR System (Ilumia, CA, US) with SYBR Green PCR Master Mix (Promega, US). Q-PCR was run with a 15 μL reaction mixture containing 1.0 μL template DNA, 0.1 μL of forward and reverse primers respectively, 6.3 μL ddH_2_O and 7.5 μL SYBR Green PCR Master Mix. The primers Arch-amoAF/Arch-amoAR were used for the quantification of archaeal *amoA* gene, with a PCR condition starting at 95 °C for 3 min, followed by 45 cycles of 30 s at 95 °C, 45 s at 53 °C and 1 min at 72 °C. The primers Nxr-f27/Nxr-r617 were used for Nitrospira *nxrB* gene quantification, with a PCR condition starting at 95 °C for 2 min, followed by 40 cycles of 45 s at 95 °C, 45 s at 62 °C and 45 s at 72 °C. The primers amoA-1F/amoA-2R were used for bacterial *amoA* gene quantification, with a PCR condition starting at 95 °C for 3 min, followed by 40 cycles of 15 s at 95 °C, 1 min at 55 °C, and 1 min at 72 °C. The Plasmid Mini Preparation Kit (Tiagen, China) was used to extract plasmids containing the targeted gene fragments from *E.coli* hosts. The extracted plasmid DNA with tenfold serial dilutions was used to obtain the standard curves. Samples and standards were determined in triplicate and average values were calculated. Melt curves generating in each assay were applied to check the specificity of the amplified products.

### Nucleotide data of high throughput deposition

The nucleic acid sequences of high throughput used in this study was deposited in the National Omics Data Encyclopedia (NODE). The accession number of archaeal *amoA* gene, bacterial *amoA* gene and *nxrB* gene were OEP002783, OEP002784, OEP002785, respectively.

## Results

### Time series of DIN during incubations

**Fig. 3:**
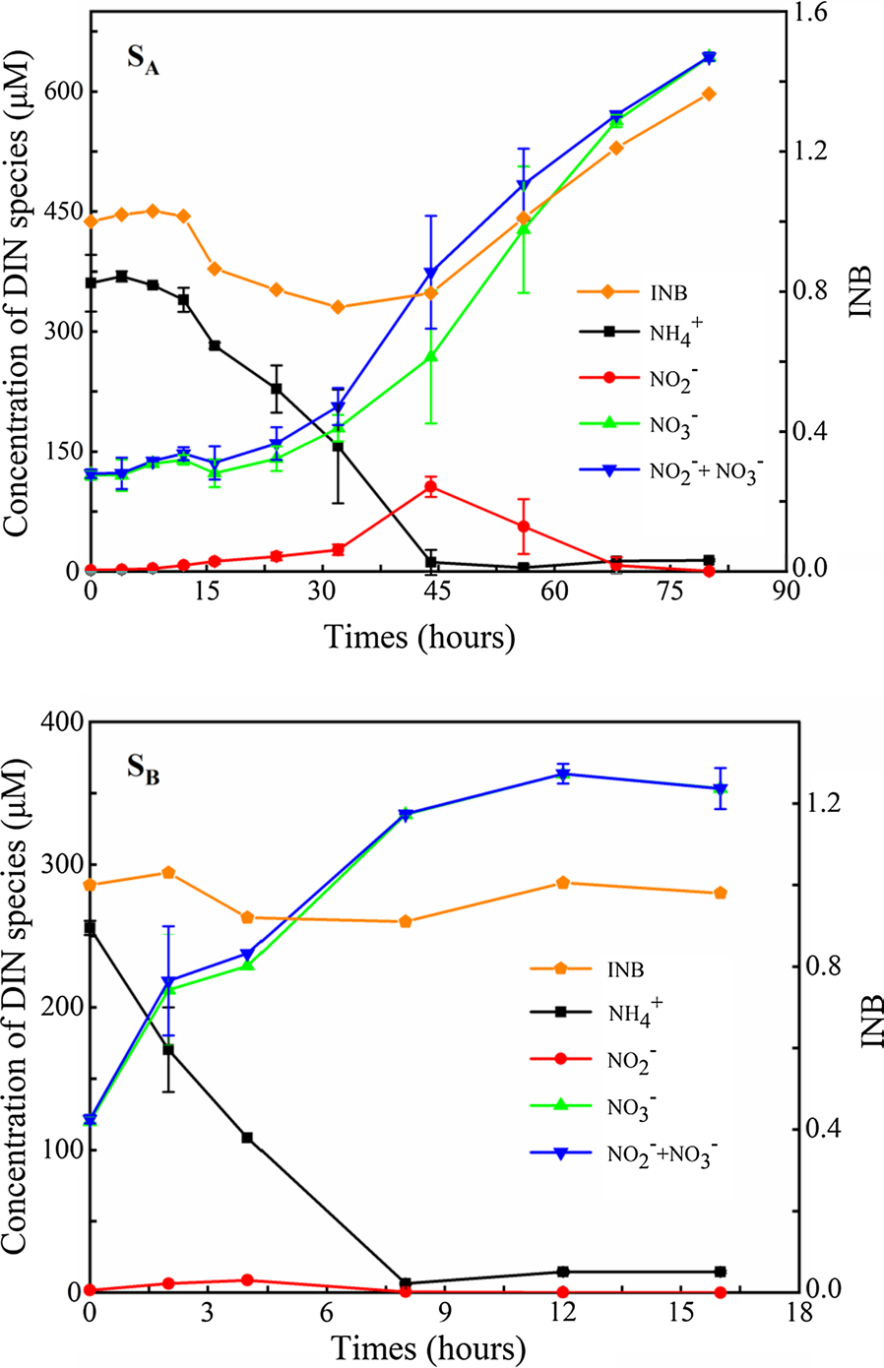
Time series for the concentrations of ammonium, nitrite and nitrate in the activated sludge of the aeration tank in Xinfeng WWTPs. Error bars represent standard variation (SD, n = 3)

Time series of ammonia, nitrite, and nitrate concentrations during the incubations of sample A (S_A_) and sample B (S_B_) were displayed in Fig. 3.

In the incubation of sample A, nitrate began to increase in accordance with a reduction in ammonia. Nitrate of sample A did not reach its maximum concentration until the nitrite was below the detection limit. Correspondingly, the variation in NO_x_^−^ concentrations (nitrite and nitrate) presented the same trend to that of NO_3_^−^. The concentration of nitrite increased slowly, reaching a maximum level (105.88 µmol L^−1^) at 44 h, then it began to decrease. As INB < 1.0, it was indicated that the nitrogen was lost owing to diffenent possible ways such as denitrification, anammox and assimilation by phytoplankton. When INB > 1.0, it was resulted form the organic mineralization in the incubation (Hong et al. 2018). The INB value of sample A firstly decreased from 1.0 to 0.8 at the initial stage of incubation and then began to increase from 0.8 to 1.37, suggesting that assimilation and mineralization co-occurred in the period. Also, the change was closely related to the accumulation of nitrite.

The trend of DIN variation in sample B was inclined to be different from that of sample A. The ammonia concentration gradually decreased at a relatively stable rate, reaching a minimum concentration (6.34 µmol L^−1^) at 8 h. No remarkable nitrite accumulation was observed in the incubation period, suggesting that the rate of NO was equal to or stronger than the rate of AO. The nitrite concentration increased slowly, reaching a maximum level (8.66 µmol L^−1^) at 4 h and then reducing to very low level at 8 h. Due to the absence of nitrite accumulation, the time series of nitrate variation followed a similar trend to that of the sum of nitrate and nitrite. In the incubation of sample B, the INB value remained stable at about 1.0.

### Ammonia oxidation and nitrite oxidation rates

For samples A and B, the Boltzmann fitted curves for both V_a_ and V_n_ exhibited similar patterns with an initial lag phase, an exponential growth phase, and a plateau phase. Based on the fitted curve data, the average V_a_ and V_n_ values in sample A were calculated as 6.51 µmol L^−1^ h^−1^ and 6.52 µmol L^−1^ h^−1^ respectively, while the average V_a_ and V_n_ values in sample B were 14.48 µmol L^−1^ h^−1^ and 14.59 µmol L^−1^ h^−1^, respectively (Fig. 2).

The equations and curves of instantaneous V_a_ and V_n_ values (Fig. 1b and d) were established based on the first derivative of the fitted curves (Fig. 1a and c). The dynamic characteristics of V_a_ and V_n_ was clearly exhibited in the instantaneous rate curves in the incubations of sludge samples. In the incubation of sample A, both instantaneous V_a_ and V_n_ were inclined to present the characteristics of first increasing and then decreasing. After a slow period of growth lasting for about 20 h, V_a_ and V_n_ began to increase at a faster rate and reached their maximum levels of 11.11 µmol L^−1^ h^−1^ and 13.68 µmol L^−1^ h^−1^ by 50 h, then gradually declined until the end of the incubation period (Fig. 1). However, the instantaneous V_a_ and V_n_ curves of sample B were significantly different from those in sample A. Both instantaneous V_a_ and V_n_ maintained a declining trend in the incubation period, resulting in the initial V_a_ and V_n_ values their maximum values were 7.6 µmol L^−1^ h^−1^ and 11.11 µmol L^−1^ h^−1^, respectively.

### The abundance of microbial communities in nitrification

**Fig. 4:**
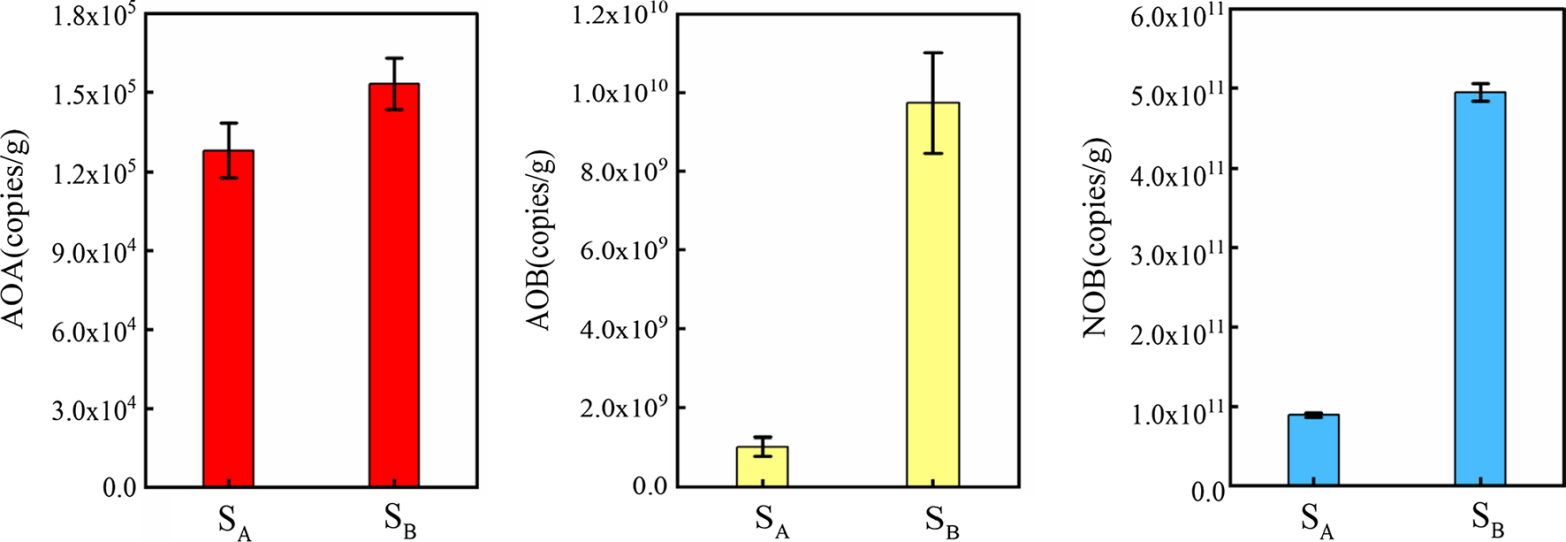
Abundances of AOA, AOB and NOB in the aeration tank of Xinfeng WWTPs

The abundances of AOA, AOB and NOB communities were estimated via the genes *amoA* and *nxrB* using the qPCR method. The abundance of AOA was the lowest in the microbial communities, ranging from 1.2 × 10^5^ to 1.5 × 10^5^ copies/g sludge (Fig. 4). In comparison, the abundance of AOB was 4 orders of magnitude higher than AOA, ranging from 1.0 × 10^9^–9.7 × 10^9^ copies/g sludge. However, the abundance of NOB *Nitrospira* was found to be 1–2 orders of magnitude higher than that of AOB.

### OTU-level composition of nitrification microbial communities

All the raw sequences obtained via high-throughput sequencing were subjected to quality control processing, trimming, filtering, and removing low-quality sequences to obtain a total of 489, 24,524, and 14,283 high-quality AOA, AOB and NOB functional gene sequences, respectively.

**Fig. 5:**
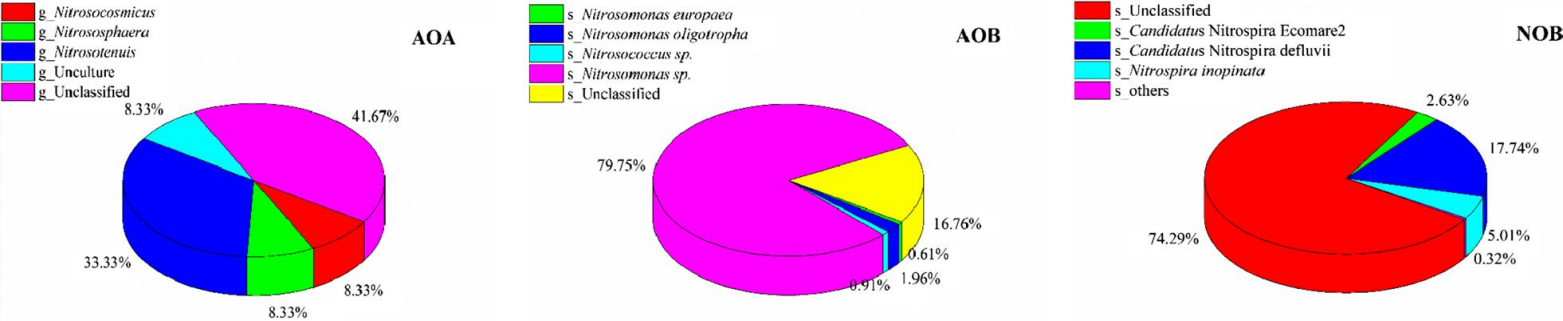
Community composition of AOA, AOB and NOB based on the dominated OTUs

The AOA, AOB and NOB community composition was shown in Fig. 5. Based on the 0.06 distance level, a total of 12 dominant OTUs were obtained for the AOA *amoA* gene. About half of these sequences were attributed to three known genera, including *Nitrosotenuis* (33.33%), *Nitrososphaera* (8.33%), and *Nitrosocosmicus* (8.33%) (Fig. 5a). Similarly, 154 dominant OTUs were obtained for the AOB *amoA* gene at the 0.13 distance level, which were assigned to four species. *Nitrosomonas sp.* was the most dominant (79.75%), followed by *Nitrosococcus sp.* (0.91%), with only a small portion classified as specific species, including *Nitrosomonas europaea* (0.61%) and *Nitrosomonas oligotropha* (1.96%) (Fig. 5b). Among the NOB community, 1556 dominant OTUs were obtained at the 0.06 distance level, the majority of which were not affiliated with any known genera (74.29%), while the remaining sequences were classified as *Candidatus* Nitrospira defluvii (17.74%), *Nitrospira inopinata* (5.01%), *Candidatus* Nitrospira Ecomare2 (2.63%), and others (0.32%) including *Nitrospira japonica* and *Nitrospira marina* (Fig. 5c).

## Discussion

Despite sample A and B were collected at different times from the anammox-inoculated wastewater treatment tank, the variation of time series of DIN in two samples exhibited similar trends. Sample A and sample B were collected in different seasons with similar temperature. Therefore, sampling times may be a major element affecting the nitrification activity of activated sludge in this study. The nitrification activity of sample A was weaker than that of sample B, which may be related to the various sampling times of activated sludge. In the incubation period of sample A, nitrite was accumulated that associated with substrates provided by the AO process. However, NOB had the highest abundance among the nitrification community which represented that the oxidation of nitrite may be delayed while NO activity may need induction with sufficient nitrite concentrations. In the incubation of sample B, almost no nitrite accumulation was observed, indicating a high NO capability and highlighting the different active function states in sample B compared to sample A.

Results showed that the instantaneous rates of V_a_ were initially higher than V_n_ for both sample A and B, then the instantaneous rates of V_n_ gradually exceeded that of V_a_ in the incubation (Fig. 1). The instantaneous rates of both V_a_ and V_n_ were higher in sample B than sample A, with the maximum rate of V_a_ in sample B being fivefold higher than sample A, while the maximum rate of V_n_ in sample B was more than threefold higher than that of sample A. Furthermore, after the maximum rates were achieved, the reduction in rates observed in sample B were faster than that of sample A, which were consistent with the observed rates of NO, which tended to be higher than that of AO in sample B (Fig. 3).

The abundance of AOB performed higher than that of AOA in sludge, indicating that AOB played a dominant role in AO (Fig. 4). This result was similar to the majority of consequences in former researches that suggested the AOB *amoA* gene abundance was typically 2–4 orders of magnitude higher than that of AOA (Jin et al. 2010; Limpiyakorn et al. 2011; Wells et al. 2009; Yapsakli 2010). The abundance of AOB has been reported to be higher than AOA in NH_3_^+^ rich environments (Yang et al. 2016), with *Nitrosomonas* AOB usually detected in this circumstance (Ke et al. 2012; Wang et al. 2013). However, some previous studies have suggested that the dominance of AOMs may not directly be related to their contribution to ammonia oxidation, they showed the process was mediated mainly by AOA though AOB was more abundant (Pan et al. 2018a, b; Niu et al. 2013). In the current study, the copy numbers of NOB in this study have exceeded many previous studies from various environmental habitats such as Chinese WWTPs (Pan et al. 2018a, b), Drinking Water Treatment Plants sludge (DWTPs) (Flower et al. 2018), freshwater recirculating aquaculture systems (Bartelme et al. 2017), and soils (Hu et al. 2017).

The abundances of all three nitrification microbial communities in sample A were lower than that of sample B as well as the abundances of AOB and NOB in sample B were tenfold and fivefold higher than sample A, respectively (Fig. 4). The most previous studies focused on ammonia-oxidizing microbes, neglecting the important role of nitrite-oxidizing microbes (Gao et al. 2018; Zhang et al. 2018; Meng et al. 2017; Ali et al. 2015). The results of the present study show that the activated sludge had strong nitrite oxidation activity besides ammonia oxidation activity.

Except the cultured three known genera of AOA, the rest of its community were composed of uncultured or unclassified genera because of the limited size of 16S reads and various databases via ambiguous classification. Some new AOA species existing in the system was possible which was similar to the study of nitrifying archaeal in the north Pacific (Semedo et al. 2021). As we known, the *Nitrosomonas* was dominant species in AOB. Among the *Nitrosomonas* bacterial community, all species were unclassified species except for *Nitrosomonas europaea* and *Nitrosomonas oligotropha*. *Nitrosomonas* was one of the most common genera found to be responsible for AO in WWTPs (Coskuner et al. 2002; Siripong et al. 2007; Wang et al. 2012). These findings were consistent with previous results for Xinfeng WWTPs (Yang et al. 2020a, b), suggesting that *Nitrosomonas* was the only AOB genus exhibiting significant levels of enrichment in the incubation. NOB *Nitrospira* was found to be the main contributor of NO in this study. However, there are many bacterial genera known to be capable of NO, with the known nitrite oxidizing genera including *Nitrospira*, *Nitrobacter*, *Nitrotoga*, *Nitrococcus*, *Nitrospina*, and *Nitrolancea* (STéPHANIE et al. 1994; Alawi et al. 2009; Daims et al. 2016). In particular, previous studies have suggested that *Nitrobacter* was a driver of NO in biological WWTPs (Gieseke et al. 2003). Then, the later researches found both *Nitrobacter* and *Nitrospira* existed in municipal WWTPs (Siripong et al. 2007). *Nitrospira* played a key role in the NOB community in activated sludge and nitrifying fluidized bed reactors especially in WWTPs of nitrite limitation due to the competitive advantage provided by the periplasmic nitrite oxidoreductase (NXR) (Yang et al. 2020a, b; Hovanec et al. 1996; Hovanec et al. 1998; Juretschko et al. 1998; Schramm et al. 1998; Wagner et al. 1996; Yang et al. 2021; Daims et al. 2016). But *Nitrobacter sp.* were found to be dominant rather than *Nitrospira sp.* in the nitrifying systems of coke WWTPs and in bioreactors treating high strength wastewater (Cho et al. 2014; Kim et al. 2011a, b; Figuerola et al. 2010; Kim et al. 2011a, b). It was also suggested that NOB *Nitrotoga* was generally detected in WWTPs at low temperatures (Alawi et al. 2009). Therefore, the actual abundance of NOB in Xinfeng WWTPs may be much higher than the level reported in the present study owing to the similar temperatures of sampling times and the same sampling location.

### Implication for regulating the nitrification process

Anammox has been identified in recent decades as an efficient and cost-effective pathway for N removal using NH_4_^+^ and NO_2_^−^ as substrates to generate N_2_ gas without the production of N_2_O in anaerobic environments (Li et al. 2020; Yang et al. 2020a, b; Meng et al. 2021). Although anammox plays an important part in the Xinfeng anammox-inoculated WWTPs, it couldn’t replace the effect of the combined network composed of nitrification, denitrification and anammox pathways in N removal. In ammonium-rich wastewater treatment, microbial nitrification is an indispensable process, which was a significant limiting-rate step in the nitrogen removal process, connecting mineralization and nitrogen loss processes (Kuypers et al. 2018; Zhang et al. 2018; Zhou et al. 2015; Yang et al. 2021). Through the nitrification process, microorganisms can transform ammonia into nitrite and nitrate (Yang et al. 2020a, b; Yang et al. 2018; Wang et al. 2021), providing effective substrates for both the anammox and denitrification processes, ensuring effective nitrogen removal by the whole treatment system (Additional file 1).

Notably, a certain concentration of nitrite helps to maintain efficient anammox processes in nitrification. However, high of NO was observed, with the activity exceeding that of AO, inhibiting nitrite accumulation (Fig. 2). Subsequently, the anammox process would be weakened. Therefore, appropriate inhibition of NOB activity in the treatment system would be an alternative method for improving the anammox activity in anammox-inoculated WWTPs, avoiding an increase in operational costs and a reduction in nitrogen removal efficiency caused by the lack of nitrite accumulation. Overall, establishing methods to regulate the nitrification process is an important issue and the composition and activity of NOB community in anammox-inoculated WWTPs should be pay more attention in future studies.

## Supplementary Information

**Additional file 1.** **Table S1** The monitoring data of Xinfeng WWTPs on May 13th. **Table S2** The monitoring data of Xinfeng WWTPs on October 12th. **Figure S1** The sewage treatment process flow chart of Xinfeng. (Wang et al. 2021).

## Data Availability

The data of nucleic acid sequences were deposited in the National Omics Data Encyclopedia (NODE) with accession number OEP002783, OEP002784 and OEP002785 for archaeal *amoA* gene, bacterial *amoA* gene and *nxrB* gene, respectively.
