# Supplementary material for: Nitrification mainly driven by ammonia-oxidizing bacteria and nitrite-oxidizing bacteria in an anammox-inoculated wastewater treatment system
Source: AMB Express. 2021 Nov 27;11:158. doi: 10.1186/s13568-021-01321-6 (PMC8627542; doi:10.1186/s13568-021-01321-6)
Supplement: Supplementary file 1 — Additional file 1. Table S1 The monitoring data of Xinfeng WWTPs on May 13th. Table S2 The monitoring data of Xinfeng WWTPs on October 12th. Figure S1 The sewage treatment process flow chart of Xinfeng. (Wang et al. 2021). [file 13568_2021_1321_MOESM1_ESM.docx]

**Supplementary Information**

**AMB Express**

**Nitrification mainly driven by ammonia-oxidizing bacteria and nitrite-oxidizing bacteria** **in an** **anammox-inoculated wastewater treatment system**

Jing Lu^a,b^, Yiguo Hong^a*^, Ying Wei^b^, Ji-Dong Gu^c^, Jiapeng Wu^a^, Yu Wang^a^, Fei Ye^a^, Jih-Gaw Lin^d^

^a^ Key Laboratory for Water Quality and Conservation of the Pearl River Delta, Ministry of Education, Institute of Environmental Research at Greater Bay, Guangzhou University, Guangzhou, 510006, P.R. China.

^b^ School of Environmental Science and Engineering, Guangzhou University, Guangzhou, China

^c^ Environmental Engineering, Guangdong Technion Israel Institute of Technology, 241 Daxue Road, Shantou 515063, Guangdong, China

^d^ Institute of Environmental Engineering, National Chiao Tung University, 1001 University Road, Hsinchu City, 30010, Taiwan, China

Corresponding author: Yiguo Hong, E-mail: [yghong@gzhu.edu.cn](mailto:yghong@gzhu.edu.cn)

**Contents of this file**

Tables S1 to S2

Figure S1

**Introduction**

This document includes the sewage treatment process flow chart and the monitoring data of Xinfeng WWTPs. ( The sampling dates were May 13^th^ 2019 and October 12^th^ 2019, respectively).

**Captions:**

Table S1 The monitoring data of Xinfeng WWTPs on May 13^th^.

Table S2 The monitoring data of Xinfeng WWTPs on October 12^th^.

Figure S1 The sewage treatment process flow chart of Xinfeng. (Wang et al. 2021)

**Table S1** The monitoring data of Xinfeng WWTPs on May 13^th^.

| May 13^th^ | Ammonium (mg L^-1^) | COD  (mg L^-1^) | Nitrite  (mg L^-1^) | Nitrate  (mg L^-1^) |
| --- | --- | --- | --- | --- |
| Effluent water | - | - | - | - |
| Supernatant | 222.5 | 480 | 0 | 0 |
| Influent water | - | - | - | - |
| Sedimentation tank A | 5 | 480 | 0 | 183 |
| Sedimentation tank B | 22.5 | 1200 | 0 | 997 |

**Table S2** The monitoring data of Xinfeng WWTPs on October 12^th^.

| Oct. 12^th^ | Ammonium  (mg L^-1^) | COD  (mg L^-1^) | Nitrite  (mg L^-1^) | Nitrate  (mg L^-1^) |
| --- | --- | --- | --- | --- |
| Effluent water | 55.6 | 176 | 6 | 58.5 |
| Supernatant | 247 | 800 | 0 | 23 |
| Influent water | 265 | 3360 | 0 | 22 |
| Sedimentation tank A | 109 | 200 | 7 | 85 |
| Sedimentation tank B | 45 | 224 | 3 | 56 |


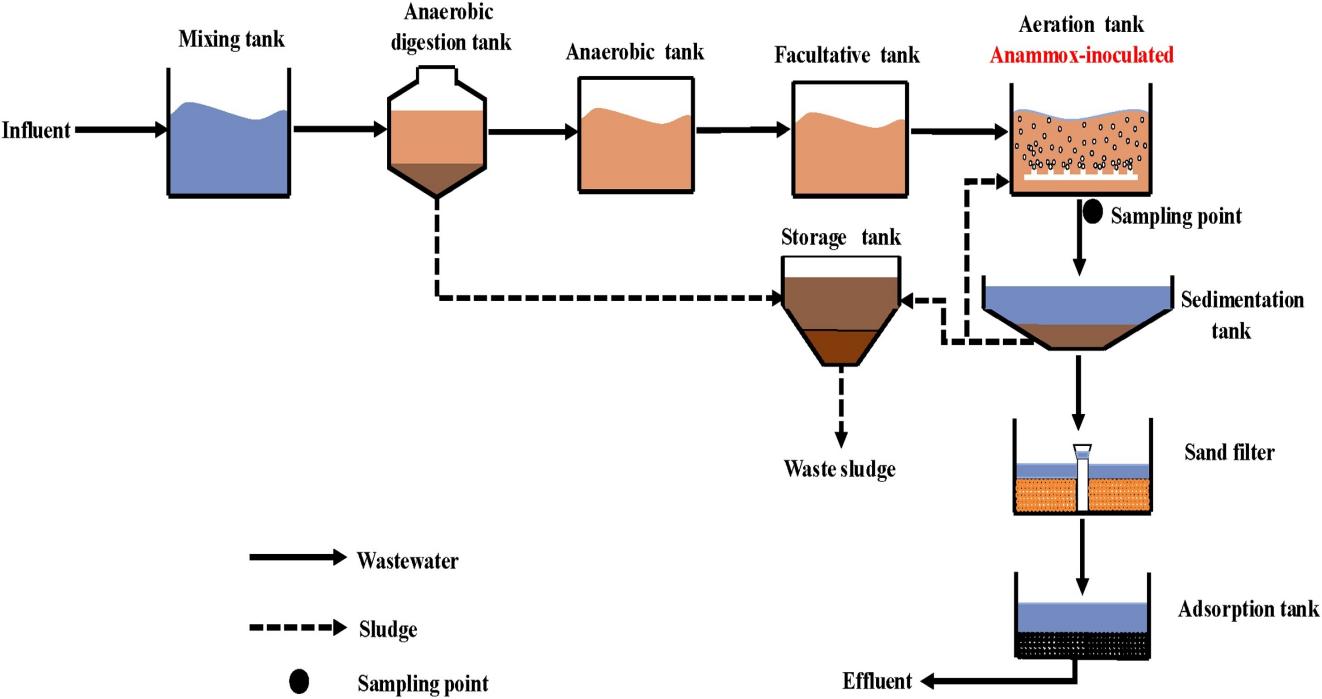


**Figure S1** The sewage treatment process flow chart of Xinfeng. (Wang et al. 2021)

**References**

Wang L, Hong Y, Gu J-D, Wu J, Yan J, Lin J-G (2021) Influence of critical factors on nitrogen removal contribution by anammox and denitrification in an anammox-inoculated wastewater treatment system. J Water Process Eng 40:101868
